# Identification and Validation of Autophagy-Related Gene Nomograms to Predict the Prognostic Value of Patients with Cervical Cancer

**DOI:** 10.1155/2021/5583400

**Published:** 2021-06-25

**Authors:** Jinqun Jiang, HongYan Xu, YiHao Wang, Hai Lu

**Affiliations:** ^1^Department of Clinical Laboratory, Yuebei People's Hospital, Shaoguan, Guangdong Province 512026, China; ^2^Department of Gynaecology, Yuebei People's Hospital, Shaoguan, Guangdong Province 512026, China; ^3^The Second Clinical College of Guangzhou University of Chinese Medicine, Guangzhou, Guangdong Province 510282, China; ^4^The Second Affiliated Hospital of Guangzhou University of Chinese Medicine, Guangzhou, Guangdong Province 510282, China; ^5^Department of Breast Diease, Guangdong Provincial Hospital of Chinese Medicine, Guangzhou University of Chinese Medicine, Guangzhou, Guangdong Province 510282, China

## Abstract

Autophagy is a process of engulfing one's own cytoplasmic proteins or organelles and coating them into vesicles, fusing with lysosomes to form autophagic lysosomes, and degrading the contents it encapsulates. Increasing studies have shown that autophagy disorders are closely related to the occurrence of tumors. However, the prognostic role of autophagy genes in cervical cancer is still unclear. In this study, we constructed risk signatures of autophagy-related genes (ARGs) to predict the prognosis of cervical cancer. The expression profiles and clinical information of autophagy gene sets were downloaded from TCGA and GSE52903 queues as training and validation sets. The normal cervical tissue expression profile data from the UCSC XENA website (obtained from *GTEx*) were used as a supplement to the TCGA normal cervical tissue. Univariate COX regression analysis of 17 different autophagy genes was performed with the consensus approach. Tumor samples from TCGA were divided into six subtypes, and the clinical traits of the six subtypes had different distributions. Further absolute shrinkage and selection operator (LASSO) and multivariable COX regression yielded an autophagy genetic risk model consisting of eight genes. In the training set, the survival rate of the high-risk group was lower than that of the low-risk group (*p* < 0.0001). In the validation set, the AUC area of the receiver operating characteristic (ROC) curve was 0.772 for the training set and 0.889 for the verification set. We found that high and low risk scores were closely related to TNM stage (*p* < 0.05). The nomogram shows that the risk score combined with other indicators, such as G, T, M, and N, better predicts 1-, 3-, and 5-year survival rates. Decline curve analysis (DCA) shows that the risk model combined with other indicators produces better clinical efficacy. Immune cells with an enrichment score of 28 showed statistically significant differences related to high and low risk. GSEA enrichment analysis showed the main enrichment being in *KRAS* activation, genes defining epithelial and mesenchymal transition (EMT), raised in response to the low oxygen level (hypoxia) gene and NF-*k*B in response to TNF. These pathways are closely related to the occurrence of tumors. Our constructed autophagy risk signature may be a prognostic tool for cervical cancer.

## 1. Introduction

Cervical cancer is the most common malignancy of the female reproductive tract; it is diagnosed in millions of women each year and causes 300,000 deaths worldwide [1]. Among women, this disease ranks fourth in mortality and morbidity [2]. Since early symptoms of cervical cancer are not obvious, late lymph node metastasis is common, leading to poor prognosis. The current treatment method is surgery or chemical radiation [3]. Therefore, revealing the molecular mechanism of cervical cancer would provide new targets for its diagnosis and improve patient prognosis.

Autophagy is a process of self-phagocytosis. The hallmark of autophagy is the formation of autophagosomes, where lysosomes wrap cytoplasmic proteins or organelles and achieve self-renewal [4]. Autophagy is also one of the most important cytoplasmic recycling mechanisms [5]. This process is closely related to the carcinogenesis, with cancer cells relying on systemic autophagy in their cytoplasm and in the host to sustain growth [6], providing the necessary nutrients and raw materials [7].

Many studies have demonstrated the important role of autophagy in cervical cancer. Therefore, autophagy can be used as a target for the treatment of cervical cancer. Studies have shown that Tubeimoside-I (TBM), as a new lethal autophagy lysosomal inducer, can induce autophagy accumulation and may enhance the therapeutic effect of chemotherapeutic drugs on cervical cancer [8]. Moreover, autophagy promotes paclitaxel resistance in cervical cancer cells [9]. Autophagy also plays an important role in preventing cisplatin-induced apoptosis of cervical cancer cells, suggesting that inhibition of autophagy may improve cisplatin chemotherapy [10]. However, while these studies have revealed the role of autophagy in the occurrence and development of cervical cancer and its relationship with various tumor drugs, few studies have examined the prognostic role of autophagy in cervical cancer.

In this study, we built eight autophagy gene risk models and used these to predict the prognosis of cervical cancer overall survival in the TCGA queue, verified in the GSE52903 queue. All had very good diagnostic performance and further revealed the relationship between high- and low-risk and immune infiltration and high-risk biological function prognosis.

## 2. Materials and Methods

### 2.1. Data Set Selection

We downloaded the mRNA expression profiles and clinical information of cervical cancer patients from the TCGA and GEO databases, respectively. Samples with incomplete clinical information and gene expression values below 0 were deleted. In the TCGA database, there are few normal tissue data matched with cervical cancer, and the total number of cases is only three. Thus, we downloaded the expression values of the normal cervix from the UCSC Xena database (https://xenabrowser.net). The GSE52903 data set includes 17 normal tissues and 55 tumor tissues. The clinical information of the two data sets is shown in Supplementary Table 1. The list of 232 autophagy genes comes from HADb (human Bite database, https://www.autophagy.lu/).

### 2.2. Identifying Differentially Expressed ARGs

We used the Wilcoxon test to analyze the difference between 232 autophagy genes based on the limma package [11]. The cut-off value was selected as log2-fold change (FC) > 1 and an adjusted *p* value of <0.05.

### 2.3. Enrichment Analysis Based on Univariate COX Analysis of Differential ARGs

To study the function of the differential univariate COX autophagy genes, we used the “clusterProfiler” package [12] to visualize gene ontology (molecular function, cellular composition, and biological function) and KEGG pathways.

### 2.4. Genotyping of Differential COX Autophagy

We used the “ConsensusClusterPlus” [13] package to cluster the differential COX genes, used the “survival” package to see the survival curve of the typing and used the “heatmap” package to visualize the relationship between the autophagy gene and each clinical feature. Finally, the “ggplot2” [14] package was used to show the relationship between different clinical features and genotyping.

### 2.5. Construction and Verification of Prognostic Autophagy Gene Signatures

We identified the autophagy genes associated with the prognosis of cervical cancer by univariate COX regression, LASSO regression, and multivariate COX regression. In summary, univariate COX regression identified the genes associated with prognosis, followed by further reduction of the genes using LASSO, multivariate COX regression, and Using *R* packet “glment” to construct LASSO regression model. Then we constructed a risk model with the final prognostic autophagy gene, and the risk score was calculated as follows:

(1) $$\begin{array}{c} \text{risk score}=\sum_{i=1}^{n}{\text{Coef}}_{i}\times x_{i}, \end{array}$$

where Coef is the coefficient and *x* is the expression level of the autophagy gene.

Using this risk score, we were able to divide 259 cervical cancer patients with complete clinical data from the TCGA training set and 46 cervical cancer patients with complete clinical information from the GSE52903 validation set into high- and low-risk groups. We then looked at whether there were differences between the high- and low-risk groups in TCGA and in the clinical subtypes of GEO. A Kaplan–Meier curve was used to observe the prognosis of the high- and low-risk groups, and the receiver operating characteristic (ROC) curve was used to assess the specificity and sensitivity of the prognostic model. The *R* software package “SurvivalROC” is used to draw the receiver operating characteristic (ROC) of the subjects and the area under the curve (AUC). Then we associate the risk assessment with clinical characteristics and use univariate and multivariate COX regression to analyze whether the risk score is an independent prognostic factor. Finally, the differences in clinical subtypes between the high- and low-risk groups were demonstrated in TCGA and GEO.

### 2.6. Building of the Nomogram

A nomogram, also known as a nomograph, is a graphical method of evaluating complex functions. In this study, a nomogram was constructed using risk scores and clinical features to assess patient survival. This method visually represents the value range of different variables and their contribution to the value of risk. A nomograph was drawn with *R* packages “rms” and “foreign,” which are more intuitive than building a table of coefficients. The accuracy of the model was then evaluated using standard curves and combined clinical characteristic indicators (ROC).

### 2.7. Relationship between Risk Score, Autophagy-Related Genes, and Immunity

The immune gene set, consisting of 782 genes, was used to predict the abundance of 28 immune cells in 305 cervical cancer tumor tissues based on ssGSEA method and implemented with the GSVA R package, with an immune genetic set from https://www.cell.com/cms/10.1016/j.celrep.2016.12.019/attachment/f353dac9-4bf5-4a52-bb9a-775e74d5e968/mmc3.xlsx [15]. We next looked to see whether there was a statistical difference between the 28 immune cells in the high- and low-risk groups, calculated the correlation between prognostic autophagy genes and 28 immune cells, selected the cell types with *p* < 0.05, and visualized them with ggplot2 *R* package.

### 2.8. Drawing the DCA Decision Curve

The *R* function “stdca” [16] was used to draw the DCA decision curve based on the COX regression model to evaluate the clinical utility.

### 2.9. Biological Function of High- and Low-Risk Groups

The Pi *R* package (https://pi314.r-forge.r-project.org/), based on the GSEA method, was used to assess high- and low-risk groups. The biological function of enrichment for the MsigdbH gene was set at https://www.gsea-msigdb.org/gsea/index.jsp.

### 2.10. Statistical Analysis

All statistical analyses were performed using *R* software (version 3.6.3). The Wilcoxon test was used for the difference in autophagy genes between tumor and normal tissue, and both the Wilcoxon and Kruskal–Wallis tests were used for the correlation between high- and low-risk score and clinical subtypes of the TCGA test set and GEO verification set. The correlation method used between autophagy gene and immune cells was the Spearman method. *p* < 0.05 was considered statistically significant.

## 3. Results

### 3.1. Identification of ARGs

**Figure 1:**
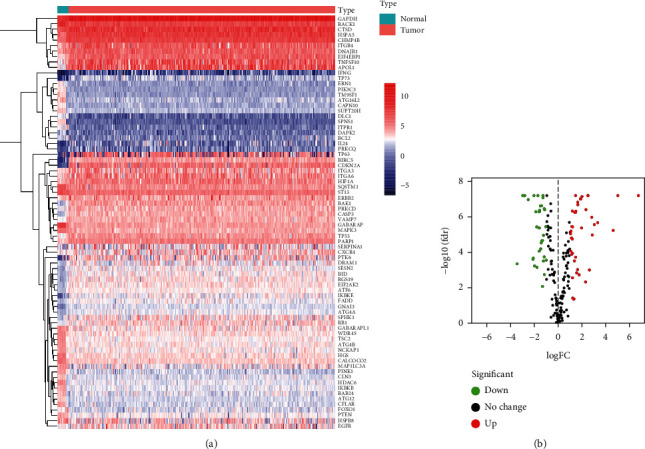
Identification of differentially expressed autophagy genes. (a) Heatmap of differential autophagy genes in cervical cancer. (b) Volcano map of differential autophagy genes, absolute log2-fold change (FC) > 1, and adjusted *p* value < 0.05 were used as screening criteria for differential genes.

A total of 84 different autophagy genes were found in tumor tissues and normal tissues (combined with *GTEx*), including 44 upregulated and 40 downregulated genes (Figures 1(a) and 1(b)).

### 3.2. Functional Annotation of Prognostic Autophagy Genes

**Figure 2:**
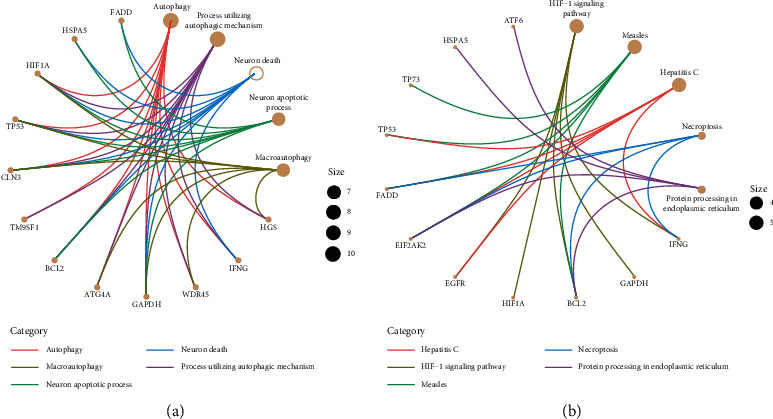
Functional enrichment of prognostic autophagy genes. (a) Analysis of the top-five most important gene ontologies. (b) Analysis of the top-five most important KEGG pathways.

The differential autophagy genes were obtained as 18 genes using the univariate COX regression method. Gene ontology enrichment analysis showed that these 18 genes were mainly enriched in autophagy, macroautophagy, the neuron apoptotic process, and the neuron death process utilizing an autophagic mechanism (Figure 2(a)). KEGG pathway enrichment analysis showed that these genes were mainly enriched (Figure 2(b)).

### 3.3. Identification of Cervical Cancer Subtypes Based on Prognostic Autophagy Genes

**Figure 3:**
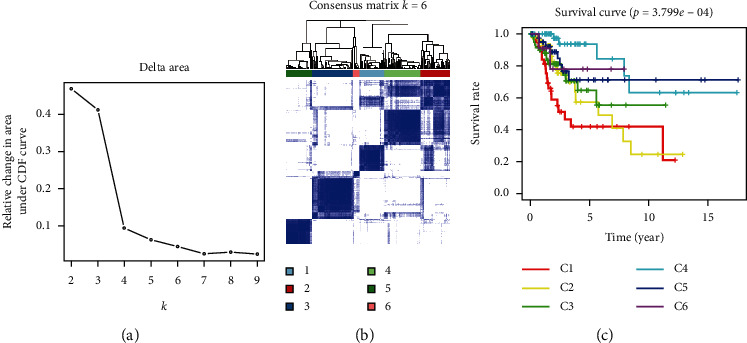
Prognostic autophagy, genotyping, and genotyping survival analysis. (a) CDF delta area curves for all samples. (b) When *K* = 6 (1 = C1, 2 = C2, 3 = C3, 4 = C4, 5 = C5, and 6 = C6), cervical cancer patients were divided into subtype matrix. (c) Survival curve analysis of six subtypes.

**Figure 4:**
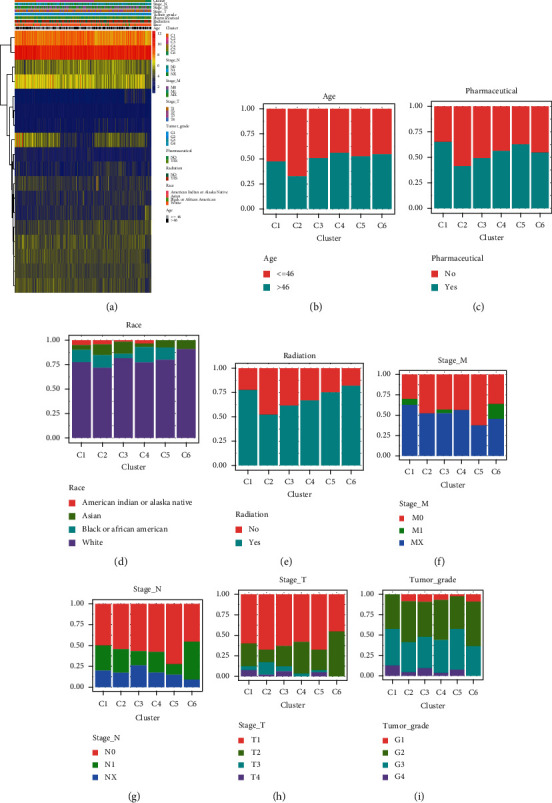
Correlation between six subtypes and clinical characteristics. (a) The heat map shows the expression values, subtypes, and clinical characteristics of 18 autophagy genes. Red indicates high expression and blue indicates low expression. (b) Distribution of age in subtypes. (c) Distribution of pharmaceutical treatments among subtypes. (d) Distribution of different races in subtypes. (e) Distribution of radiation therapy in subtypes. (f) Distribution of M stages in subtypes. (g) Distribution of N stages in subtypes. (h) Distribution of T stages in subtypes. (i) Distribution of Grade in subtypes.

SigClust analysis results showed that, among all clusters, the curve was flat when the consensus cluster was *K* = 6 (Figure 3(a)). Meanwhile, 261 tumor samples were divided into six molecular subtypes (Figure 3(b)). Survival analysis showed that subtype C4 had a better survival rate compared to others (*p* < 0.001) (Figure 3(c)). A heat map was constructed, showing the relationship between the expression of 18 autophagy genes and six subtypes and various clinical characteristics (Figure 4(a)). With age as the median, the C2 subtype had the lowest proportion of ages greater than 46 years old (Figure 4(b)). Subtype C1 had the highest percentage of pharmaceutical intervention (Figure 4(c)). Among races, Caucasian had the highest proportion among all subtypes. There were only Asian and Caucasian cases in subtype C6 (Figure 4(d)). The highest proportion undergoing radiotherapy was found in subtype C2 (Figure 4(e)). In subtype C5, the proportion without distant metastasis was the highest, and in subtype C6, the proportion with distant metastasis was higher than that of other subtypes (Figure 4(f)). In terms of regional lymph node involvement, similar results were found with or without distant metastasis. In subtype C5, the proportion of no lymph node involvement was the highest compared with other subtypes, while in subtype C6, the proportion of involvement of a few lymph nodes was the highest (Figure 4(g)). In terms of primary tumors, the proportion of T1 in subtype C6 was the lowest compared to other subtypes, with the proportion of T2 being the largest, and only within these two subtypes (Figure 4(h)). For tumor grade, the proportion of G2 in subtype C6 was the highest (Figure 4(i)).

### 3.4. Construction and Validation of a Risk Model Based on Eight Autophagy Genes

**Figure 5:**
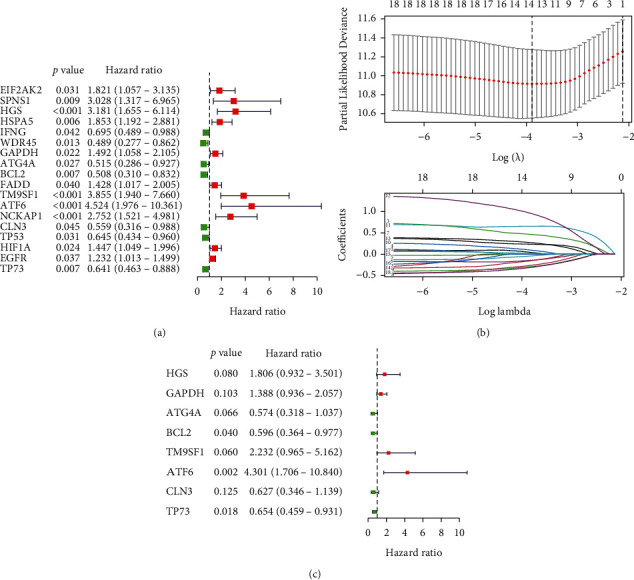
Identification of eight autophagy prognostic genes. (a) Univariate COX regression analysis of differential autophagy genes. (b) LASSO regression analysis of 17 autophagy genes. (c) Multivariate COX regression analysis of autophagy genes.

In the training set from TCGA, the 18 autophagy genes obtained by the univariate COX regression method (Figure 5(a)) were further reduced by the LASSO regression method (Figure 5(b)). The eight autophagy genes (Table 1) were used for the construction of the risk signature based on the multivariate COX regression method (Figure 5(c)):

(2) $$\begin{array}{c} \text{risk score}=0.5914*\left(\text{expression level of }HGS\right)+0.238*\left(\text{expression level of }GAPDH\right)\\ +\left(-0.556\right)*\left(\text{expression level of }ATG4A\right)+\left(-0.518\right)*\left(\text{expression level of }BCL2\right)\\ +0.8038*\left(\text{expression level of }TM9SF1\right)+1.459*\left(\text{expression level of }ATF6\right)\\ +\left(-0.466\right)*\left(\text{expression level of }CLN3\right)+\left(-0.425\right)*\left(\text{expression level of }TP73\right). \end{array}$$

**Figure 6:**
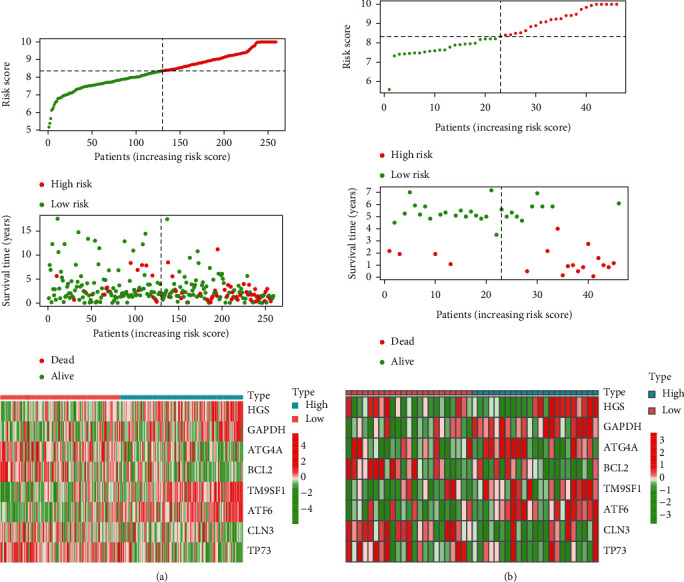
Construction and validation of risk signatures of eight autophagy prognostic genes. The relationship between risk score (top), risk scores and survival time (middle), risk score and gene expression in training set TCGA (a) and validation set GSE52903 (b). The black dotted line represents the median of the risk score.

**Figure 7:**
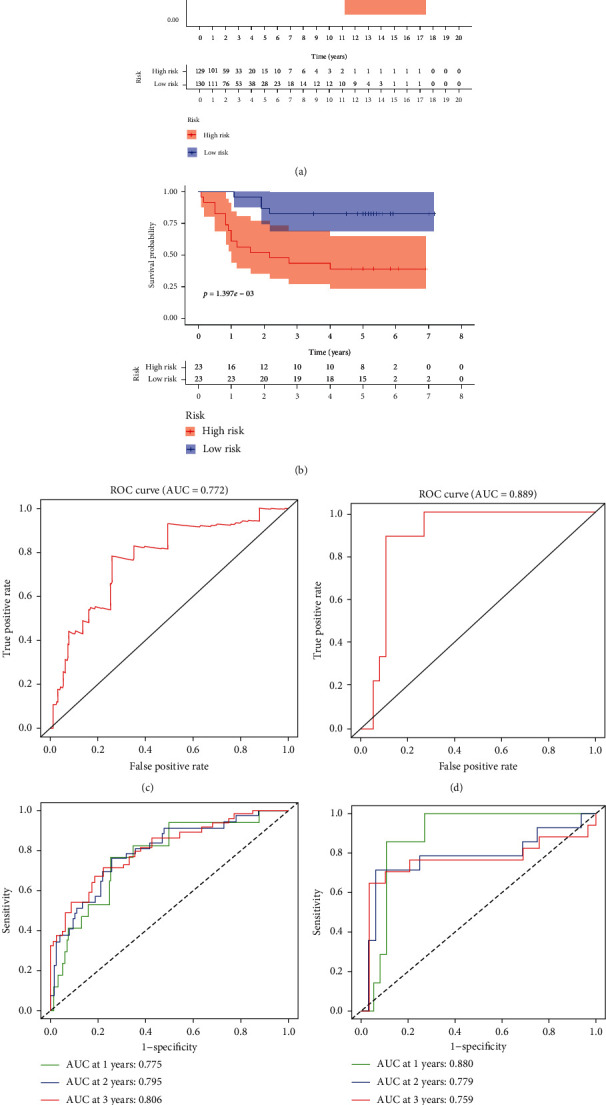
Kaplan–Meier survival analysis and ROC curve analysis risk model. Kaplan–Meier survival curves of the high- and low-risk groups were found in TCGA (a) and GSE52903 (b). ROC curve analysis of TCGA (c) and GSE52903 (d). The ROC curve analysis was used to predict 1, 3, and 5 years in TCGA (e) and GSE52903 (f).

We then calculated each patient's risk score and divided them into high- and low-risk groups according to the median of the scores in the TCGA cohort of the training set (Figure 6(a)) and the GSE52902 cohort of the verification set (Figure 6(b)). The patient's survival status, survival time, and autophagy gene expression are shown in Figures 6(a) (TCGA) and 6(b) (GSE52903). As demonstrated, the number of patients dying gradually increased as the risk score increased. Survival analysis showed that the high-risk group had a worse prognosis in TCGA (*p* < 0.0001) (Figure 7(a)), with a consistent result of *p* < 0.01 in the GES52903 validation set (Figure 7(b)). ROC curve analysis results showed that the AUC value in TCGA was 0.772 (Figure 7(c)) and 0.889 for GES52903 (Figure 7(d)), and the AUC values for 1, 2, and 3 years were 0.775, 0.795, 0.806 and 0.880, 0.779, 0.759, respectively (Figure 7(e)). These findings suggest that the risk score has a good predictive ability for overall survival.

### 3.5. Univariate and Multivariate COX Regression Analysis of Risk Score

**Figure 8:**
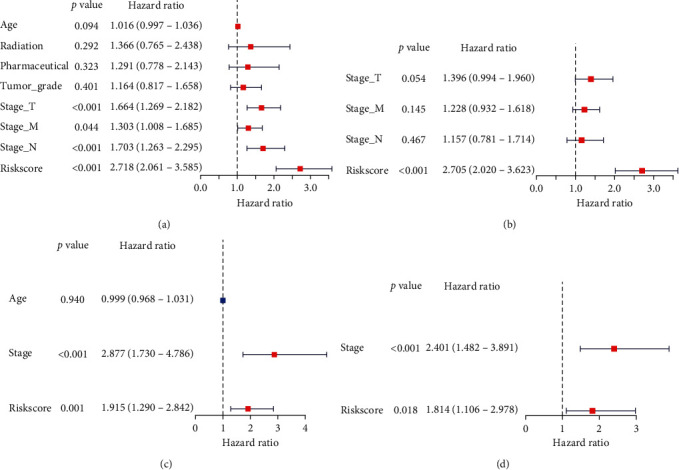
Univariate and multivariate COX regression analysis of clinical characteristics and risk score for overall survival. (a, b) Univariate and multivariate COX regression analysis of clinical characteristics and risk scores for overall survival in TCGA. (c, d) Univariate and multivariate COX regression analysis of clinical characteristics and risk scores for overall survival in the GEO.

As shown in Figure 8, in the TCGA cohort, univariate COX regression analysis showed that T staging, M staging, N staging, and risk score were independent prognostic factors and could predict overall survival (stage_T: *p* < 0.001). In the GEO cohort, univariate COX regression analysis showed that stage and risk score could predict overall survival (stage: *p* < 0.001 and risk score: *p* < 0.001; Figure 8(c)). In the TCGA cohort, multivariate COX regression analysis showed that only risk score could predict the overall survival (risk score: *p* < 0.001, Figure 8(b)). In the GEO cohort, both stage and risk score could predict the overall survival (stage: *p* < 0.001 and risk score: *p* = 0.018; Figure 8(d)). The above data indicate that the risk score can predict the overall survival rate independently of any clinical trait.

### 3.6. Relationship between Risk Score and Clinical Traits

**Figure 9:**
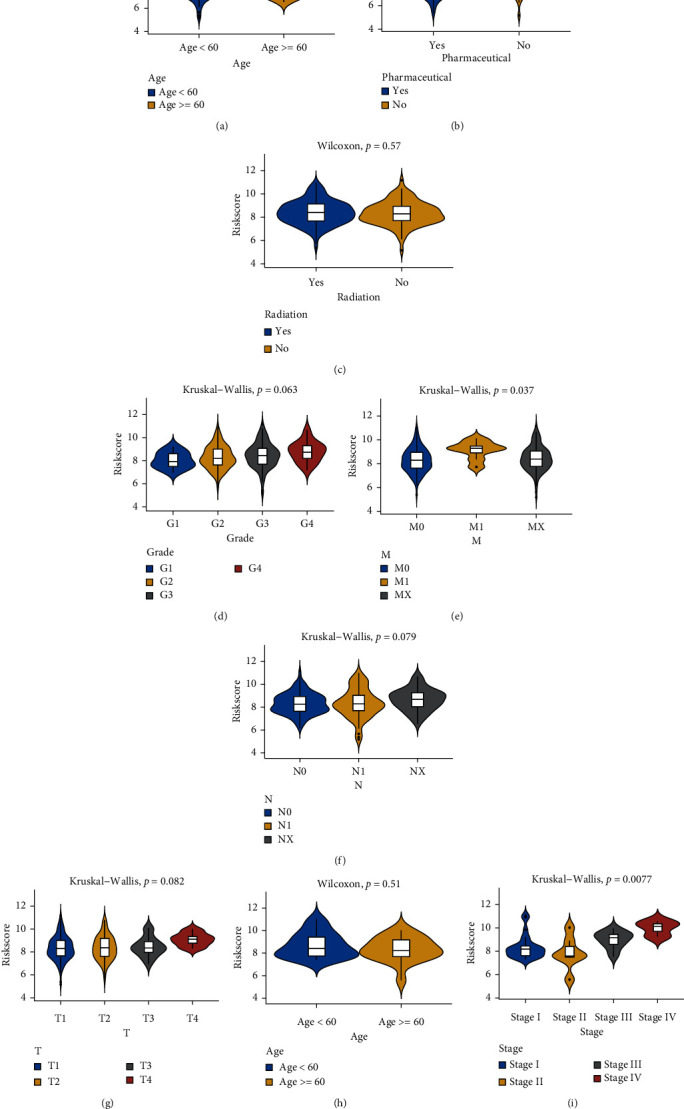
Differences in risk scores of various clinical traits. (a) In TCGA, the difference in risk scores with age as the median value, *p* = 0.27. (b) Differences in risk scores in the pharmaceutical in TCGA, *p* = 0.55. (c) Difference in risk score between receiving radiation therapy and not receiving radiation therapy in TCGA, *p* = 0.57. (d) Risk scores differed among different grading groups in TCGA, *p* = 0.063. (e) Differences between different M staging groups in TCGA, *p* = 0.037. (f) Differences between different N staging groups in TCGA, *p* = 0.079. (g) Differences between different T staging groups in TCGA, *p* = 0.082. (h) Difference in risk scores with age as the median value in GES52903, *p* = 0.51. (i) Differences between staging groups in GSE52903, *p* = 0.0077.

As shown in Figure 9, the risk score in the TCGA cohort showed statistically significant differences in metastasis (Figure 9(e)), while in the GSE52903 cohort, the risk score was statistically significant in tumor stage (Figure 9(i)). In the TCGA cohort, age and whether the patient had received pharmaceutical or radiotherapy were not significant. This suggests that the risk score is closely related to the tumor stage.

### 3.7. Construction of Nomogram

**Figure 10:**
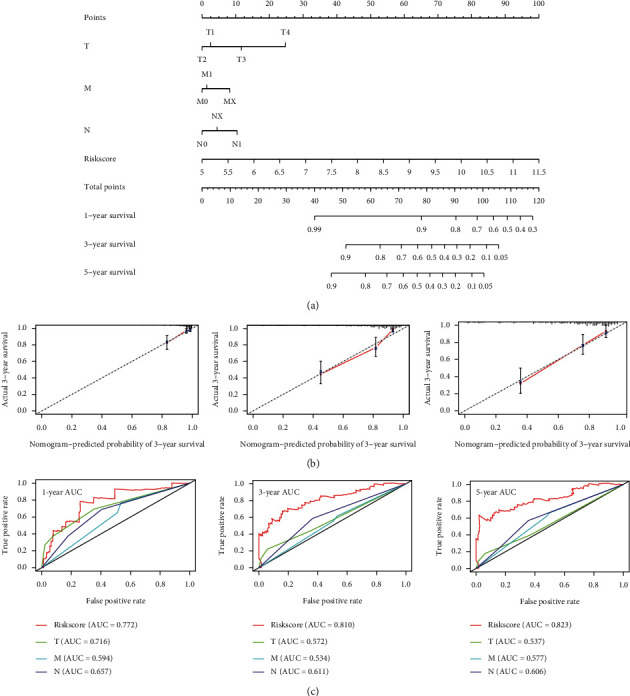
Constructing a nomograph in cervical cancer to predict overall survival. (a) Risk score and age, G, T, M, and N nomograph. (b) Standard curve for predicting 1-, 3-, and 5-year overall survival rate. (c) Multi-index ROC curve analysis of risk score and clinical characteristics.

Based on the risk score, G, T, M, and N staging, a nomograph was constructed (Figure 10(a)). The total score based on the above indicators could be predicted for each patient for 1-, 2-, and 5-year survival rates. A standard curve was used to evaluate the predictive ability of the nomograph. As shown in Figure 10(b), the 1-, 3-, and 5-year curve levels overlapped well with the standard curve. At the same time, the multi-index ROC curve analysis results of combined clinical traits showed that the risk score AUC areas of 1-, 3-, and 5-year survival were all higher than other clinical characteristics, which were 0.772, 0.810, and 0.823, respectively (Figure 10(c)). These results indicate that the risk scoring model has good predictive ability.

### 3.8. DCA Curve Drawing

**Figure 11:**
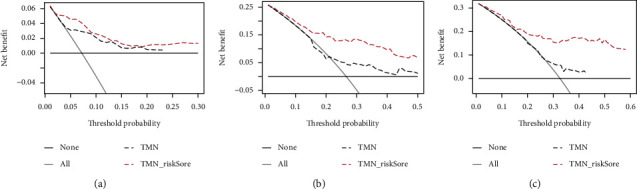
Construct the DCA curve. (a–c) The DCA curve of a model with a joint risk score of 1 year (10A), 3 years (10B), and 5 years (10C) and a model with TMN stage.

Considering the clinical utility of the risk model, we drew a DCA curve. As shown in Figure 11, the model combined with the risk score was more beneficial than the model with TNM stage. Figures 11(a)–11(c) show the 1-, 3-, and 5-year survival DCA curve.

### 3.9. Relationship between Risk Models Constructed by Eight Autophagy Genes, Immune Cells, and Their Biological Functions

**Figure 12:**
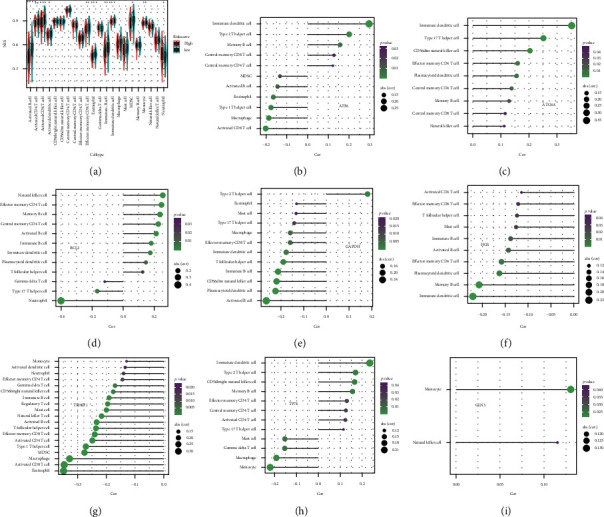
Based on the relationship between eight autophagy gene models and immune cells. (a) Analysis of the difference between the high- and low-risk groups in 28 immune cells, calculate the *p* value with Wilcoxon test and add it as an asterisk at the top of the picture. (b–i) Correlation between autophagy genes and immune cells. Spearman's correlation method was used to calculate the correlation between eight autophagy genes and immune cells, and the immune cells with *p* < 0.05 were selected.

**Figure 13:**
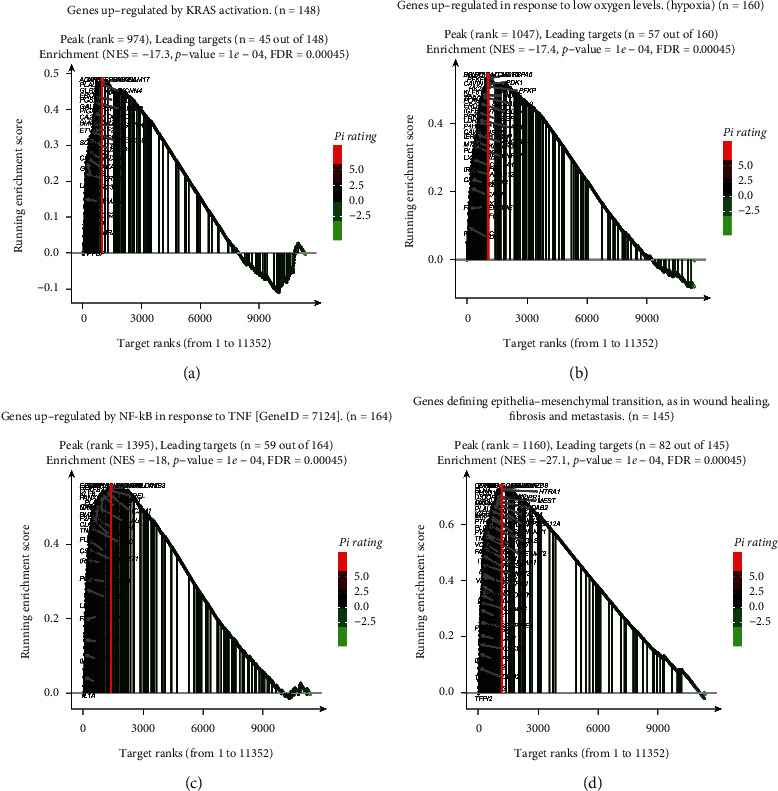
GSEA analysis for high- and low-risk groups. (a) Mainly enriched in *KRAS* activation. The genes marked in black are the dominant genes. (b) Mainly enriched in genes upregulated in response to low oxygen levels (hypoxia). The genes marked in black are the dominant genes. (c) Mainly enriched in the genes regulated by NF-*k*B in response to TNF. The genes marked in black are the dominant genes. (d) Mainly enriched in the genes defining epithelial–mesenchymal transition, as in wound healing, fibrosis, and metastasis. The genes marked in black are the dominant genes.

As shown in Figure 12(a), high- and low-risk scores differed in most immune cells except for the following: CD56^bright^ natural killer cells, CD56^dim^ natural killer cells, central memory CD4^+^ T-cells, effector memory CD8^+^ T-cells, macrophages, mast cells, gamma delta T-cells, memory B-cells, natural killer cells, and natural killer T-cells. Figure 12(b)to 12(i) shows the correlations between *HGS*, *GAPDH*, *ATG4A*, *BCL2*, *TM9SF1*, *ATF6*, *TP73*, and *CLN3* genes and different immune cells. We then performed GSEA on the high- and low-risk scores. As shown in Figure 13, the high-risk group was mainly enriched in genes related to *KRAS* activation, hypoxia, NF-*k*B in response to TNF, EMT, wound healing, fibrosis, and metastasis. These biological functions are closely related to tumorigenesis.

## 4. Discussion

The latest 2020 cancer statistics show that the global mortality rate of cervical cancer patients is relatively high for countries in transition (12.4 vs 5.2 per 100,000) [17]. Due to the advent of the internet, sexual knowledge has also become more available, which also exacerbates the occurrence of cervical cancer and produces younger occurrences. Of importance to note, traditional TNM staging cannot identify early cervical cancer, which leads to worse prognosis. Late-stage cervical cancer is linked to poor prognosis, putting forth a need for a more effective method to predict disease earlier. Recently, a prognostic model constructed based on autophagy-related genes has attracted the attention of researchers; for example, in patients with colon cancer [18], head and neck squamous cell carcinoma [19], esophageal cancer [20], gastric cancer [21], glioma [22], hepatocellular carcinoma [23] and others, it has been proven that a prognostic model constructed by autophagy-related genes can predict the prognosis of tumor survival.

Many studies have shown that autophagy is involved in multiple signaling pathways and affects the occurrence and development of cervical cancer [24–28]. Studies have shown that inhibitors of the autophagy gene *ATG4* can inhibit autophagy, enhance the cytotoxicity of chemotherapy drugs, and achieve the purpose of killing tumor cells [29]. Autophagy gene *BCL2* can be used as a therapeutic target of some drugs for cervical cancer [30]. *TM9SF1* can bind to the estrogen receptors, regulate the epithelial-mesenchymal transformation of cancer cells, and promote tumor metastasis [31]. *ATF6* is also closely related to the occurrence of colorectal cancer [32]. *TP73* is a member of the p53 transcription factor family. Owing to its low mutation rate, p53 has become an ineffective target for most tumors [33]. Recent studies have shown that ferredoxin reductase regulates the expression of *TP73* by binding to iron-binding proteins, thereby regulating aging and tumor inhibition [34]. Steroid lipofuscosis 3 (*CLN3*) is abnormally expressed in hepatocellular carcinoma and can promote tumor progression and metastasis [35].

In this study, we combined the normal cervical expression profile data of *GTEx* with data from TCGA to extract the expression data of all autophagy genes. We obtained 84 differentially expressed autophagy genes determined by single-factor COX regression analysis. For the 18 autophagy genes related to prognosis, the functional enrichment of these genes mainly enriched terms such as autophagy, apoptosis, and the HIF-1 signaling pathway, which are closely related to the occurrence of tumors [36, 37]. At the same time, 18 genotypes were classified into six subtypes, and the distribution of each clinical trait in each subtype was not the same. Next, we used LASSO regression and multifactor COX regression methods to obtain the final eight autophagy genes for use in constructing a prognosis model for cervical cancer patients. Meanwhile, 18 genotypes were classified into six subtypes, and the distribution of each clinical trait in each subtype was not the same. Next, we used LASSO regression and multifactor COX regression methods to obtain the final seven autophagy genes for constructing a prognostic risk model. We found that in the high-risk group, prognosis was poor, tumors were relatively large, there were more organs involved, and there were more distant metastases and lymph node involvement. The advantage of using a nomogram is the transformation of complex regression equations into a simple visualization graphs, making the results of prognostic models readable and widely used in clinical applications [38–41]. In this study, the risk score, age, grade, and stage were constructed to predict the 1-, 2-, and 3-year survival probability of cervical cancer patients. The 1-, 3-, and 5-year AUC values were all higher than age, classification, and staging. Since the nomogram was a predictive model, the decision curve analysis (DCA) method was used to solve the clinical utility problem and solve the clinical practicality of the nomogram. DCA is also widely used in various fields, such as predicting the prognosis of lung cancer [42], sentinel lymph node metastasis of skin melanoma [43], and the prognosis of left atrial enlargement in degenerative mitral regurgitation [44]. In this study, after adding the risk score signature, patients benefited more from 1-, 3-, and 5-year survival. Immune infiltration is also a hot topic of research. Studies have found that the combination of immune checkpoint inhibitors and cisplatin anticancer drugs can enhance the therapeutic effect of cervical cancer [45, 46]. In this study, the immune enrichment score of most immune cells in the high-risk group was lower than that in the low-risk group, and the immune activity of the high-risk group was found to be suppressed, which is more conducive to the proliferation of tumor cells. GSEA results showed that the high-risk group was mainly enriched for genes involved in *KRAS* activation, hypoxia, NF-*k*B in response to TNF, EMT, wound healing, fibrosis, and metastasis. Studies have shown that hypoxia is related to the poor prognosis of tumor patients [47]. EMT is the main driver of tumor cell metastasis [48]. These biological functions can lead to poor prognosis and more deaths in high-risk groups.

Shi et al. [49] also recently established a cervical cancer prognosis model with three ARGs. The AUC area of this model was 0.678 in the training set and 0.756 in the verification set. In our model, the AUC in the TCGA training set was 0.772, and what was even more surprising was that in the GEO verification set, the AUC was 0.889. The previous study did not explore the causes of poor survival and prognosis in the high-risk group. In our study, the relationship with immune infiltration was found in the high-risk group to be mainly enriched in genes upregulated in response to hypoxia [50–52], TNF [53, 54], EMT [55, 56], wound healing, fibrosis, metastasis, and *KRAS* activation [57, 58]. These pathways are closely related to tumorigenesis, invasion, and metastasis. In terms of immune infiltration, the low-risk group had the highest level of immune cells, indicating that the immune system was activated in the low-risk group. This also confirmed that the low-risk group had a better prognosis than the high-risk group.

This study has limitations and does not include more verification sets to verify the accuracy of the model. In the future, our team will further verify the expression of these eight autophagy genes in clinical samples and include more clinical information to validate or model. We will also further study the mechanism of these autophagy genes and the occurrence of cervical cancer.

## 5. Conclusions

The seven autophagy prognostic signatures that we have constructed may become prognostic tools for cervical cancer, whether in predictive or clinical applications, and can be used as personalized treatment strategies for cervical cancer patients.

## Figures and Tables

**Table 1:** Independent autophagy genes with signature.

Genes	Coef	HR	HR.95L	HR.95H	*p* value
*HGS*	0.591371	1.806463	0.931988	3.501451	0.079883
*GAPDH*	0.327653	1.387707	0.936265	2.056824	0.10269
*ATG4A*	−0.55565	0.573702	0.317516	1.03659	0.065636
*BCL2*	−0.51751	0.596004	0.363607	0.976936	0.04012
*TM9SF1*	0.803078	2.232402	0.965385	5.162313	0.060436
*ATF6*	1.458752	4.300591	1.706265	10.83951	0.001983
*CLN3*	−0.4664	0.627255	0.345552	1.138609	0.125217
*TP73*	−0.42531	0.653565	0.458867	0.930874	0.018427

## Data Availability

Data are available upon request to the corresponding author.

## Supplementary Materials

Supplementary Materials Table S1: clinical information of cervical cancer patients in the TCGA cohort and the GSE52903 cohort.
