# Supplementary material for: Identification and Validation of Autophagy-Related Gene Nomograms to Predict the Prognostic Value of Patients with Cervical Cancer
Source: J Oncol. 2021 Jun 25;2021:5583400. doi: 10.1155/2021/5583400 (PMC8253645; doi:10.1155/2021/5583400)
Supplement: Supplementary Materials — Table S1: clinical information of cervical cancer patients in the TCGA cohort and the GSE52903 cohort. [file 5583400.f1.docx]

Supplementary Table：

sTable 1 Clinical information of cervical cancer patients in the TCGA cohort and the GSE52903 cohort.

| **TCGA** | | | |
| --- | --- | --- | --- |
|  | **Alive** | **Dead** | **Total** |
|  | **(N=195)** | **(N=64)** | **(N=259)** |
| **race** |  |  |  |
| AMERICAN INDIAN OR ALASKA NATIVE | 4 (2.1%) | 3 (4.7%) | 7 (2.7%) |
| ASIAN | 17 (8.7%) | 4 (6.2%) | 21 (8.1%) |
| BLACK OR AFRICAN AMERICAN | 20 (10.3%) | 8 (12.5%) | 28 (10.8%) |
| WHITE | 154 (79.0%) | 49 (76.6%) | 203 (78.4%) |
| **Age (yeas)** |  |  |  |
| age <60 | 161 (82.6%) | 44 (68.8%) | 205 (79.2%) |
| age >= 60 | 34 (17.4%) | 20 (31.2%) | 54 (20.8%) |
| **radiation** |  |  |  |
| NO | 72 (36.9%) | 15 (23.4%) | 87 (33.6%) |
| YES | 123 (63.1%) | 49 (76.6%) | 172 (66.4%) |
| **pharmaceutical** |  |  |  |
| NO | 95 (48.7%) | 24 (37.5%) | 119 (45.9%) |
| YES | 100 (51.3%) | 40 (62.5%) | 140 (54.1%) |
| **grade** |  |  |  |
| G1 | 15 (7.7%) | 1 (1.6%) | 16 (6.2%) |
| G2 | 83 (42.6%) | 35 (54.7%) | 118 (45.6%) |
| G3 | 85 (43.6%) | 22 (34.4%) | 107 (41.3%) |
| G4 | 12 (6.2%) | 6 (9.4%) | 18 (6.9%) |
| **T** |  |  |  |
| T1 | 125 (64.1%) | 36 (56.2%) | 161 (62.2%) |
| T2 | 57 (29.2%) | 15 (23.4%) | 72 (27.8%) |
| T3 | 10 (5.1%) | 6 (9.4%) | 16 (6.2%) |
| T4 | 3 (1.5%) | 7 (10.9%) | 10 (3.9%) |
| **M** |  |  |  |
| M0 | 94 (48.2%) | 22 (34.4%) | 116 (44.8%) |
| M1 | 5 (2.6%) | 3 (4.7%) | 8 (3.1%) |
| MX | 96 (49.2%) | 39 (60.9%) | 135 (52.1%) |
| **N** |  |  |  |
| N0 | 123 (63.1%) | 26 (40.6%) | 149 (57.5%) |
| N1 | 39 (20.0%) | 21 (32.8%) | 60 (23.2%) |
| NX | 33 (16.9%) | 17 (26.6%) | 50 (19.3%) |
| **GEO** | | | |
|  | **Alive** | **Dead** | **Total** |
|  | **(N=28)** | **(N=18)** | **(N=46)** |
| **Age (yeas)** |  |  |  |
| age <60 | 17 (60.7%) | 11 (61.1%) | 28 (60.9%) |
| age >= 60 | 11 (39.3%) | 7 (38.9%) | 18 (39.1%) |
| **stage** |  |  |  |
| satgeI | 20 (71.4%) | 4 (22.2%) | 24 (52.2%) |
| stageII | 4 (14.3%) | 3 (16.7%) | 7 (15.2%) |
| stageIII | 4 (14.3%) | 8 (44.4%) | 12 (26.1%) |
| stageIV | 0 (0%) | 3 (16.7%) | 3 (6.5%) |
